# Dental pulp cells cocultured with macrophages aggravate the inflammatory conditions stimulated by LPS

**DOI:** 10.1186/s12903-023-03625-4

**Published:** 2023-12-09

**Authors:** Min-Ching Wang, Kuo-Wei Chang, Shu-Chun Lin, Ling-Hsin Hsu, Pei-shih Hung

**Affiliations:** 1 Division of Pediatric Dentistry, Department of Dentistry, Wan Fang Hospital, Taipei Medical University, Taipei, Taiwan; 2 Department of Dentistry, National Yang Ming Chiao Tung University, Taipei, Taiwan; 3 Department of Stomatology, Taipei Veterans General Hospital, Taipei, Taiwan; 4 Institute of Oral Biology, School of Dentistry, National Yang Ming Chiao Tung University, Taipei, Taiwan; 5 Department of Dentistry, Taipei City Hospital, Taipei, Taiwan; 6 Department of Medical Research, National Yang Ming Chiao Tung University Hospital, Yilan, Taiwan

**Keywords:** Dental pulp cells (DPCs), Macrophage, Coculture, Inflammation, LPS

## Abstract

**Background:**

Pulp inflammation is complex interactions between different types of cells and cytokines. To mimic the interactions of different types of cells in inflamed dental pulp tissues, dental pulp cells (DPCs) were cocultured with different ratios of macrophages (THP-1) or LPS treatment.

**Methods:**

DPCs were cocultured with various ratios of THP-1, then photographed cell morphology and determined cell viability by MTT assay at preset times. Total RNA was also extracted to measure the inflammation marker-*IL-6* and *IL-8* expressions by RT-Q-PCR. The DPCs and THP-1 were treated with 0.01 – 1μg/ml lipopolysaccharide (LPS) and extract RNA at preset times, and detected *IL-6* and *IL-8* expression. DPCs were cocultured with various ratios of THP-1 with 0.1 μg/mL LPS, and detected *IL-6* and *IL-8* expression after 24 and 48 h. The data were analyzed by unpaired t-test or Mann-Whitney test. Differences were considered statistically significant when *p* < 0.05.

**Results:**

THP-1 and DPCs coculture models did not suppress the viability of DPCs and THP-1. Cocultured with various ratios of THP-1 could increase IL-6 and IL-8 expressions of DPCs (*p* = 0.0056 - *p* < 0.0001). The expressions of IL-6 and IL-8 were stronger in higher ratio groups (*p* = 0.0062 - *p* < 0.0001). LPS treatment also induced IL-6 and IL-8 expressions of DPCs and THP-1 (*p* = 0.0179 – *p* < 0.0001 and *p* = 0.0189 – *p* < 0.0001, separately). Under the presence of 0.1 μg/mL LPS, DPCs cocultured with THP-1 for 24 h also enhanced IL-6 and IL-8 expression (*p* = 0.0022). After cocultured with a higher ratio of THP-1 for 48 h, IL-6 and IL-8 expressions were even stronger in the presence of LPS (*p* = 0.0260).

**Conclusions:**

Coculturing dental pulp cells and macrophages under LPS treatment aggravate the inflammatory process. The responses of our models were more severe than traditional inflamed dental models and better represented what happened in the real dental pulp. Utilizing our models to explore the repair and regeneration in endodontics will be future goals.

**Supplementary Information:**

The online version contains supplementary material available at 10.1186/s12903-023-03625-4.

## Background

Pulp inflammation is a complex process initiated by the stimulation and invasion of bacteria. Dental pulp cells secrete proinflammatory cytokines to initiate host protective events, including antibacterial, immune, and inflammatory responses, to defend against the invasion of bacteria. These inflammatory responses would pass on the signals to blood vessels, then immune cells migrate and reach the injured area and further enhance the inflammation process. Since pulp inflammation determines the fate of pulp -- repair or necrosis, we need to understand more about pulp inflammation [1].

Traditionally, pulp inflammation was studied by single-cell models induced by lipopolysaccharide (LPS), lipoteichoic acid (LTA), or physical injuries [2, 3]. However, pulp inflammation was the reactions and interactions of pulp cells, immune cells, vascular cells, nerve cells, and a few dendritic cells. The interactions between different kinds of cells need to be explored because they are crucial in this complex process. Coculture models are cell cultivation setups in which two or more different populations of cells are grown with some degree of contact between them. Hence, coculture models give us opportunities to understand better the mechanisms of pulp inflammation and other oral tissues [4, 5]. Coculture models are more representative models of human in vivo-like tissue than animal models and allow for in-depth monitoring of cell–cell interactions [6].

Considering the severity of pulp inflammation may determine the prognosis of vital pulp therapy (VPT), a better understanding of pulp biology and inflammatory process can enhance the outcomes of VPT [7–9]. Pulp inflammation is the key to pulp healing, but severe pulp inflammation may lead to the death of pulp cells. Hence, the goal of VPT may be modulating and managing inflammation appropriately instead of eliminating inflammation [10]. The null hypothesis is that coculture with macrophages is not related to pulp inflammation severity under LPS treatment. Therefore, the aim of the paper was to explore the expressions of pulp inflammation markers by coculturing dental pulp cells and macrophages under LPS treatment.

## Methods

### Culture of DPCs

This study was approved by the Institutional Review Board of Taipei Veterans General Hospital (approval no. 2017–07-023 AC). All adult participants provided written informed consent. All child participants provided written informed assent (Both child and parent). The primary human DPCs used in this study were established from a third molar from a 22-year-old female after obtaining informed consent. Pulp tissues were collected from the sectioned tooth, rinsed with PBS twice, and then minced into small fragments. The tissues were treated with trypsin/EDTA (Sigma–Aldrich, St. Louis, MO, USA) at 37 °C for 15 min and neutralized with DMEM (Corning, New York City, NY, USA) containing 10% fetal bovine serum (FBS; Biological Industries, Kibbutz Beit-Haemek, Israel), 100 U/mL penicillin, 100 mg/mL streptomycin, and 0.25 mg/mL amphotericin B (Pen-Strep-Amp antibiotics, Biological Industries). The tissues were placed onto the surface of a 100 μm cell strainer (BD Bioscience, San Jose, CA, USA) and cultured in the presence of MEM-α (Lonza, Basel, Switzerland) containing 16.6% FBS and Pen-Strep-Amp antibiotics at 37 °C in a humidified atmosphere in a 5% CO_2_ incubator [11]. In this system, single cells were able to pass through the mesh and attached to the plate below. When confluent, the cells were treated with trypsin/EDTA and passaged. All pulp cells used in this study were between passage 4 and 10.

### Culture of THP-1 and HL-60 cells

The THP-1 and HL-60 cell lines were purchased from Bioresource Collection and Research Centre (BCRC, Hsinchu, Taiwan). THP-1 was maintained in RPMI 1640 (Sigma–Aldrich) supplemented with 10% FBS, Pen-Strep-Amp antibiotics, and 0.05 mM 2-mercaptoethanol (Sigma–Aldrich) at 37 °C in a humidified atmosphere in a 5% CO_2_ incubator [12]. HL-60 was maintained in Iscove’s modified Dulbecco’s medium (Sigma–Aldrich) supplemented with 20% FBS and Pen-Strep-Amp antibiotics at 37 °C in a humidified atmosphere in a 5% CO2 incubator [13]. These cell lines were passaged every 3 to 4 days.

### The settings of each group

Seeded DPCs in 96 or 12-well plates and incubated at 37 °C in a humidified atmosphere in a 5% CO_2_ incubator overnight.

THP-1: DPCs = 1:0 – only seeded THP-1 cells in empty wells without DPCs.

THP-1: DPCs = 0:1 – only added fresh medium.

THP-1: DPCs = 1:1000 – added THP-1 equivalent to 1/1000 of the number of DPCs.

THP-1: DPCs = 1:100 – added THP-1 equivalent to 1/100 of the number of DPCs.

THP-1: DPCs = 1:10 – added THP-1 equivalent to 1/10 of the number of DPCs.

THP-1: DPCs = 1:2 - added THP-1 equivalent to 1/2 of the number of DPCs.

THP-1: DPCs = 1:1 – added THP-1 equivalent to 1/1 of the number of DPCs.

### Cell proliferation assay and cell viability assay

The alamarBlue assay was used to determine the proliferation of DPCs [14]. Briefly, DPCs were seeded in 96-well plates and incubated at 37 °C in a humidified atmosphere in a 5% CO_2_ incubator overnight. The next day, the medium was removed, and THP-1 cells with different cell ratios and 100 nM phorbol-12-myristate-13-acetate (PMA, Sigma–Aldrich) were added to THP-1 culture medium for 24 h [15]. At preset times, ten μl AlamarBlue® solution (Thermo Fisher Scientific) was added to the cultivated cells for two h and measure the fluorescence with Excitation wavelength at 530-560 nm and Emission wavelength at 590 nm by GloMax® Explorer Multimode Microplate Reader (Promega Corporation, Madison, WI, USA).

The cell viability of coculture DPCs and THP-1 cells were detected by MTT assay [11]. Briefly, DPCs were seeded in 6-well plates and then cocultured with THP-1 cells. At preset times, 1 mg/mL MTT substrate was added to the cultivated cells for 1 h and then the cell morphology was photographed.

### Expression levels of IL-6 and IL-8 by RT–qPCR

Used TRI reagent (Sigma–Aldrich) according to the manufacturer’s instructions to extract total RNA, and synthesized cDNA by a ReverTra Ace Set (TOYOBO Ideas & Chemistry, Osaka, Japan). The mRNA expression of *IL-6*, *IL-8* and *GAPDH* was examined by real-time PCR using PowerUp™ SYBR™ Green Master Mix (Thermo Fisher Scientific, Waltham, MA, USA) with gene-specific primers. The primer sequences were as follows: *IL-6*, 5′-AAC CTG AAC CTT CCA AAG ATG G-3′ (sense) and 5′-TCT GGC TTG TTC CTC ACT ACT-3′ (antisense); *IL-8*, 5′-CAT ACT CCA AAC CTT TCC ACC CC-3′ (sense) and 5′-TCA GCC CTC TTC AAA AAC TTC TCC A-3′ (antisense); *GAPDH*, 5′-AGA AGG CTG GGG CTC ATT TG-3′ (sense) and 5′-AGG GGC CAT CCA CAG TCT TC-3′ (antisense) [16]. The thermal cycling conditions were as follows: predenaturation at 95 °C for 10 min, followed by 40 cycles of denaturation at 94 °C for 15 s, and annealing and extension at 60 °C for 60 s. These reactions were carried out using a QuantStudio I system (Thermo Fisher Scientific). The comparative threshold cycle (Ct) method was used to measure relative changes in expression. The 2^-ΔΔCt^ values were used to represent the fold changes in mRNA expression between the sample groups and between the various experimental setups [17].

### Expression levels of IL-6, TNF-α and MMP-9 by ELISA

The protein levels of IL-6, TNF-α and MMP-9 in cocultured medium were detected by Lumit™ IL-6 (Human) immunoassay (Promega), Lumit™ TNF-α (Human) immunoassay (Promega) and Human MMP-9 ELISA Kit (Thermo Fisher Scientific). Briefly, DPCs were seeded in 96-well plates and incubated at 37 °C in a humidified atmosphere in a 5% CO_2_ incubator overnight. The next day, the medium was removed, and DPCs were treated with 0.1 μg/mL LPS or cocultured with THP-1 cells (THP-1: DPCs = 1:10). After 24 and 48 h, IL-6, TNF-α and MMP-9 expression were detected according to the manufacturer’s instructions.

### Statistical analysis

The data are presented as the mean ± standard deviation from at least two independent observations. The results were analyzed using GraphPad Prism (GraphPad Software Inc., San Diego, CA, USA). The Mann–Whitney test and unpaired t-test were used for comparisons between two groups, and two-way ANOVA was applied for comparisons among multiple groups. Differences were considered statistically significant when *p* < 0.05.

## Results

### THP-1 and DPCs coculture model did not suppress the growth of DPCs

**Fig. 1:**
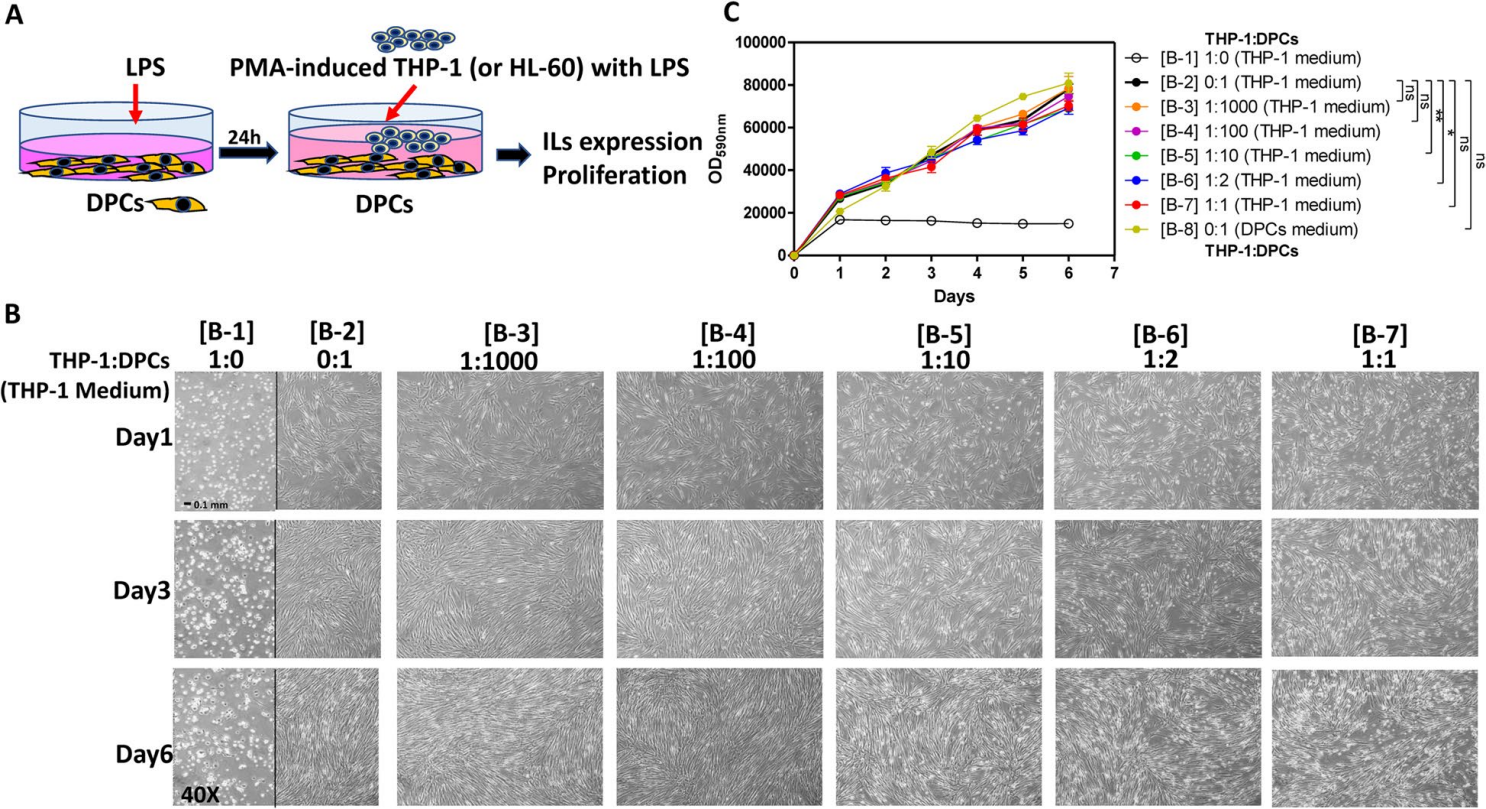
The coculture model did not suppress the growth of DPCs. **A** Illustration of the DPCs and THP-1 coculture model and LPS treatment. **B**-**C** DPCs were cocultured with different ratios of THP-1 cells with 100 nM PMA and cultivated for 6 days. [B-1] THP-1: DPCs = 1:0 (pure THP-1 in THP-1 culture medium); [B-2] THP-1: DPCs = 0:1 (pure DPCs in THP-1 culture medium); [B-3] THP-1: DPCs = 1:1000 (in THP-1 culture medium); [B-4] THP-1: DPCs = 1:100 (in THP-1 culture medium); [B-5] THP-1: DPCs = 1:10 (in THP-1 culture medium); [B-6] THP-1: DPCs = 1:2 (in THP-1 culture medium); [B-7] THP-1: DPCs = 1:1 (in THP-1 culture medium); [B-8] THP-1: DPCs = 0:1 (pure DPCs in DPCs culture medium) (**B**) The cell morphology results. THP-1 cells and DPCs did not affect the morphology of each other. Magnification × 40. **C** The growth curve of cocultured DPCs and THP-1 cells. The growth trends of DPCs of [B-2] to [B-7] groups were similar in the coculture model. The data were expressed as the mean ± SD of triplicate wells form one experiment. ns, no significance; *, *p* < 0.05; **, *p* < 0.005; compared with the [B-2] THP-1: DPCs = 0:1 (pure DPCs in THP-1 culture medium) untreated control group. Two-way ANOVA

In order to mimic clinical conditions, LPS was added to DPCs first to stimulate inflammation. Then, PMA-induced THP-1 (or HL-60) cells were added to complete the coculture model. Cell growth and mRNA expression were analyzed. The coculture model of whole this paper was depicted in Fig. 1A.

In this coculture model, THP-1 culture medium was used to maintain the survival of THP-1 cells, and 100 nM PMA was used to induce THP-1 differentiation to activated macrophage [18]. The alamarBlue and MTT assay was used to determine the growth/viability of DPCs and THP-1 cells. The growth rate of DPCs in the THP-1 culture medium was slightly slower than that in DPCs culture medium (Fig. 1C, group [B-2] and [B-8], no significance). DPCs continuously grew until 6 days of cultivation, at which point PMA-treated THP-1 cells were also alive and showed mitochondrial activity (Fig. 1B, C, and Fig. S1).

### DPCs coculture with different ratios of THP-1 cells induced different levels of ILs expression

**Fig. 2:**
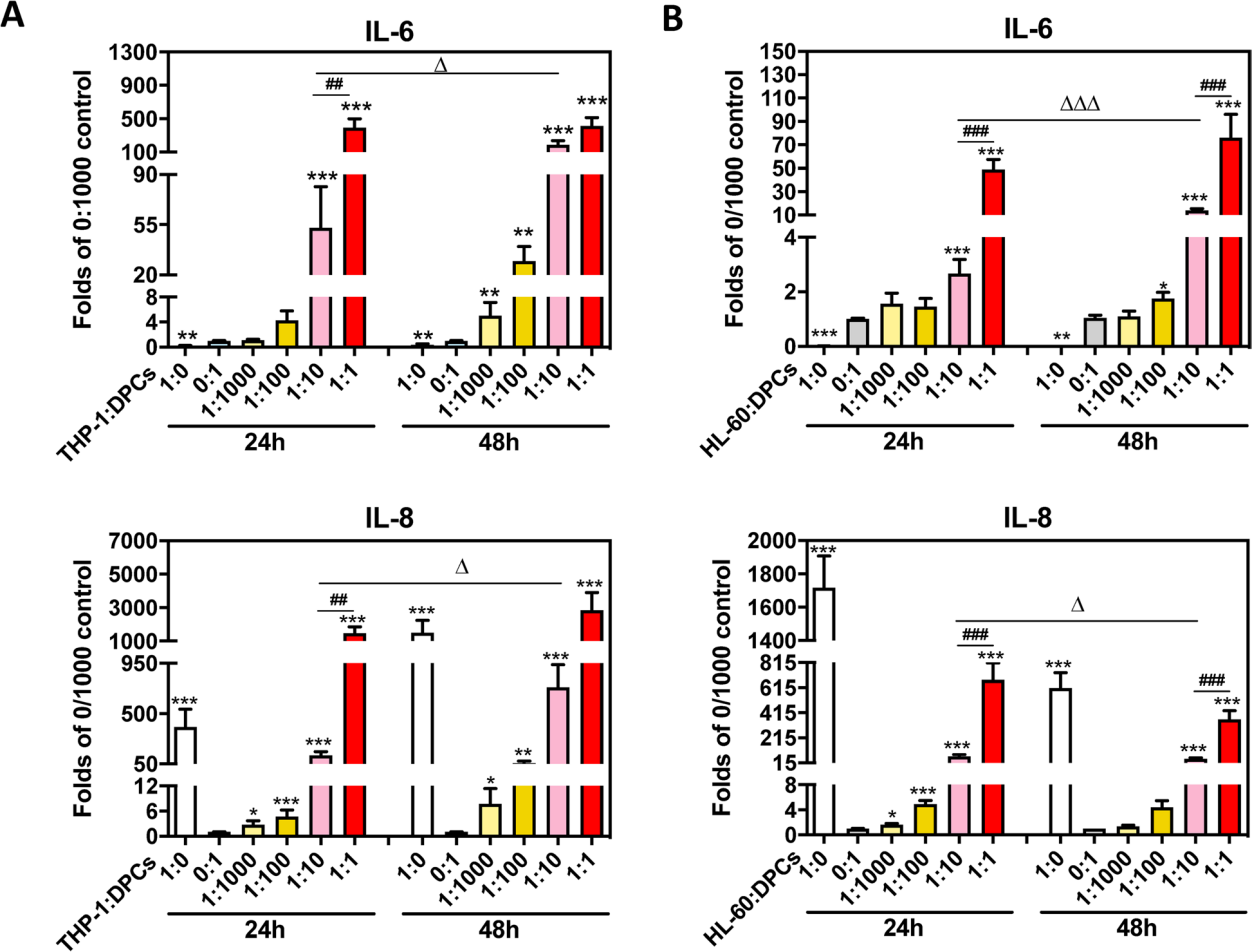
Cocultured with THP-1 cells successfully enhanced *IL-6* and *IL-8* expressions. DPCs were cocultured with different ratios of THP-1 (**A**) or HL-60 (**B**) cells with 100 nM PMA and then extracted total RNA after 24 and 48 h. *IL-6* and *IL-8* mRNA expression were determined by RT-qPCR. **A** Both THP-1: DPCs = 1:10 and THP-1: DPCs = 1:1 increased *IL-6* and *IL-8* expression at 24 and 48 h. The data were expressed as the mean ± SD of triplicates from three independent experiments. *, *p* < 0.05; **, *p* < 0.005; ***, *p* < 0.0005 compared with THP-1: DPCs = 0:1 untreated control of each time point; ##, *p* < 0.005 compared with THP-1: DPCs = 1:10 of each time point; Δ, *p* < 0.05 compare with 24 h of each ratio group. Mann–Whitney test. **B** Both HL-60: DPCs = 1:10 and HL-60: DPCs = 1:1 increased *IL-6* and *IL-8* expression at 24 and 48 h. The data were expressed as the mean ± SD of triplicates from three independent experiments. *, *p* < 0.05; **, *p* < 0.005; ***, *p* < 0.0005 compared with HL-60: DPCs = 0:1 untreated control of each time point; ###, *p* < 0.0005 compared with HL-60: DPCs = 1:10 of each time point; Δ, *p* < 0.05; ΔΔΔ, *p* < 0.0005 compare with 24 h of each ratio group. Mann–Whitney test

DPCs were cocultured with different ratios of PMA-induced THP-1 (as macrophages) or HL-60 (as neutrophils) and determined *IL-6* and *IL-8* expression. *IL-6* expression in THP-1: DPCs = 1:0 (pure THP-1 cells) was lower than THP-1: DPCs = 0:1 (pure DPCs) (*p* = 0.002, Fig. 2A). However, *IL-8* expressions in THP-1: DPCs = 1:0 was higher than THP-1: DPCs = 0:1 (*p* = 0.0002, Fig. 2A). The expressions of *IL-6* and *IL-8* in THP-1: DPCs = 1:10 and THP-1: DPCs = 1:1 at 24 and 48 h were significantly up-regulated (*p* < 0.0001 – *p* = 0.0232, Fig. 2A). The increased levels *IL-6* and I*L-8* of THP-1: DPCs = 1:1 were higher than THP-1: DPCs = 1:10 at 24 h (*p* = 0.0015, Fig. 2A). DPCs coculture with HL-60 showed similar patterns (*p* < 0.0001 – *p* = 0.0433, Fig. 2B). In addition, the expression of *IL-1α* and *IL-1β* in coculturing DPCs with THP-1 or HL-60 were similar to the results of *IL-8* (*p* = 0.0022 – *p* = 0.0286, Fig. S2).

### Compared to THP-1, LPS treatment induced earlier immune responses

**Fig. 3:**
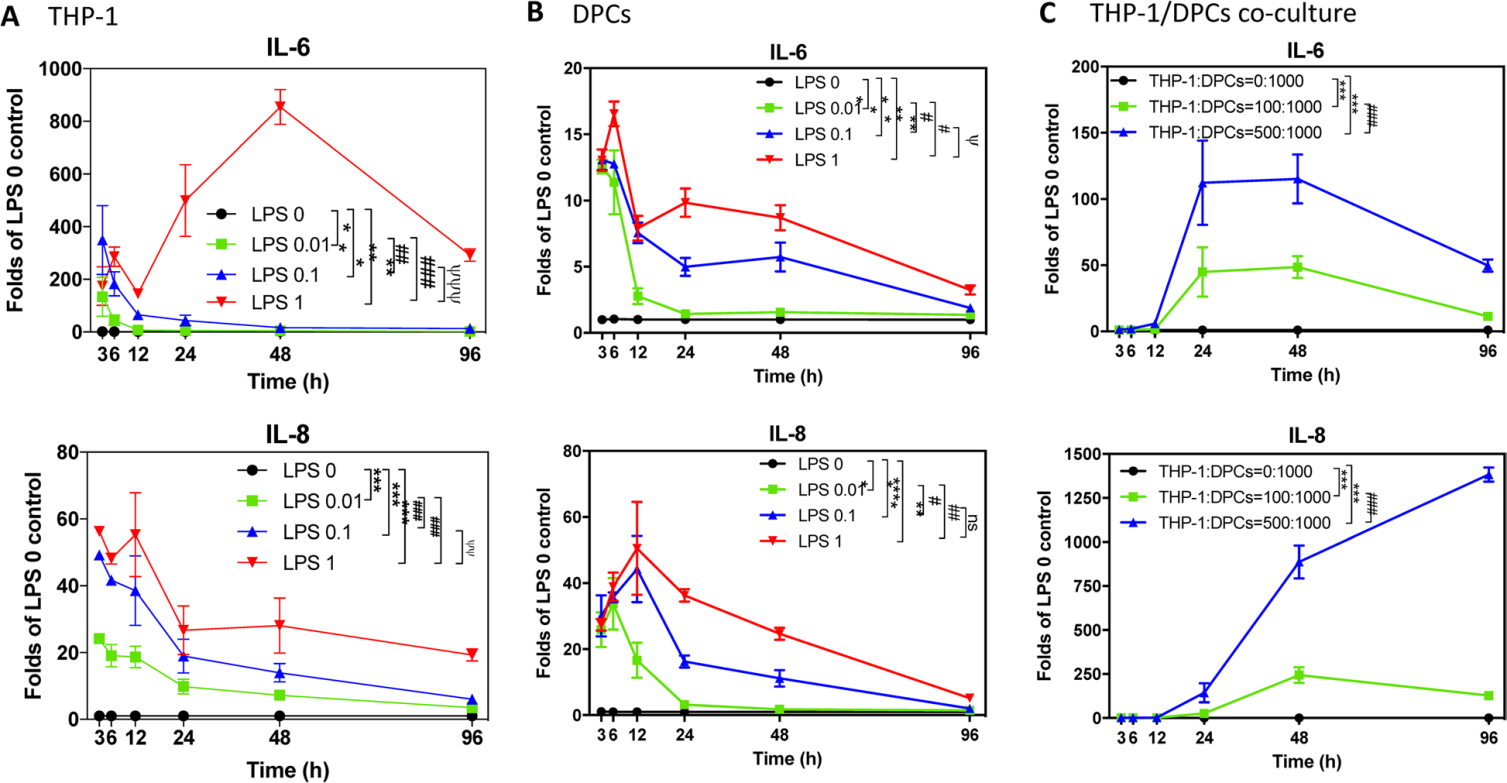
LPS and THP-1 showed different immune induction patterns in different time spans. **A** THP-1 was treated with 0, 0.01, 0.1, 1 μg/mL LPS, and total RNA was collected at 3, 6, 12, 24, 48, and 96 h. *IL-6* and *IL-8* mRNA expressions were determined by RT-qPCR. *IL-6* was up-regulated in all LPS-treated THP-1 cells. *IL-8* was up-regulated by LPS at 3–12 h, and this induction was decreased after 12 h. The data were expressed as the mean ± SD of triplicates from three independent experiments. ***, *p* < 0.0005 compared with LPS 0 μg/mL group; ###, *p* < 0.0005 compared with LPS 0.01 μg/mL group; ΨΨ, *p* < 0.005 compare with LPS 0.1 μg/mL group. Two-way ANOVA. **B** DPCs were treated with 0, 0.01, 0.1, 1 μg/mL LPS, and total RNA was collected at 3, 6, 12, 24, 48, and 96 h. *IL-6* and *IL-8* mRNA expression was determined by RT-qPCR. *IL-6* was up-regulated by LPS at 6 h, and this induction was decreased after 6 h. *IL-8* was up-regulated by LPS at 6 and 12 h, and this induction was decreased after 12 h. The data were expressed as the mean ± SD of triplicates from three independent experiments. **, *p* < 0.005; ***, *p* < 0.0005 compared with LPS 0 μg/mL group; #, *p* < 0.05; ##, *p* < 0.005 compare with LPS 0.01 μg/mL group; ns, no significance; Ψ, *p* < 0.05 compare with LPS 0.1 μg/mL group. Two-way ANOVA. **C** DPCs were cocultured with different ratios of THP-1 cells, and total RNA was collected at 3, 6, 12, 24, 48, and 96 h. *IL-6* and *IL-8* mRNA expressions were determined by RT-qPCR. *IL-6* and *IL-8* were up-regulated after 12 h, and the induction effects were decreased after 48 h. Furthermore, THP-1: DPCs = 1:2 could continuously induce *IL-8* expressions after 48 h. The data were expressed as the mean ± SD of triplicates from three independent experiments. ***, *p* < 0.0005 compared with THP-1:DPCs = 0:1; ###, *p* < 0.0005 compared with THP-1: DPCs = 1:10. Two-way ANOVA

THP-1 and DPCs were treated with 0 to 1 μg/mL LPS and determined *IL-6* and *IL-8* mRNA expression at preset times separately. In THP-1 cells, *IL-6* was up-regulated by 0.01 and 0.1 μg/mL LPS at 3 h, and this induction was decreased after 6 h. However, one ug/mL LPS treatment could up-regulate *IL-6* at 6 and 48 h (*p* = 0.0038 – *p* < 0.0001, Fig. 3A). *IL-8* was up-regulated by 0.01 to 1 μg/mL LPS at 3–12 h and this induction was decreased after 12 h (*p* = 0.0032 – *p* < 0.0001, Fig. 3A). In DPCs, with 0.01 to 1 μg/mL LPS treatment, *IL-6* was up-regulated at 6 h and then this induction was decreased till 96 h. With 0.01 μg/mL LPS treatment, the *IL-8* induction was decreased after 6 h. With 0.1 and 1 μg/mL LPS treatment, *IL-8* was up-regulated at 6 and 12 h, and then the induction effects were decreased till 96 h (*p* = 0.0189 – *p* < 0.0001, Fig. 3B). In the coculture model, DPCs were cocultured with different ratios of THP-1 cells and determined *IL-6* and *IL-8* mRNA expression at preset times. *IL-6* and *IL-8* were up-regulated after cocultured with THP-1 for 12 h, and the induction effects were kept to 48 h and decreased after 48 h. In addition, THP-1: DPCs = 1:2 could continuously induce *IL-8* expressions after 48 h till 96 h (*p* < 0.0001, Fig. 3C). The results of the whole figure demonstrated that the reciprocal inflammatory effects of DPCs and THP-1 were stronger than added up the effects of DPCs and THP-1 directly.

### DPCs cocultured with THP-1 aggravated the inflammatory conditions stimulated by LPS

**Fig. 4:**
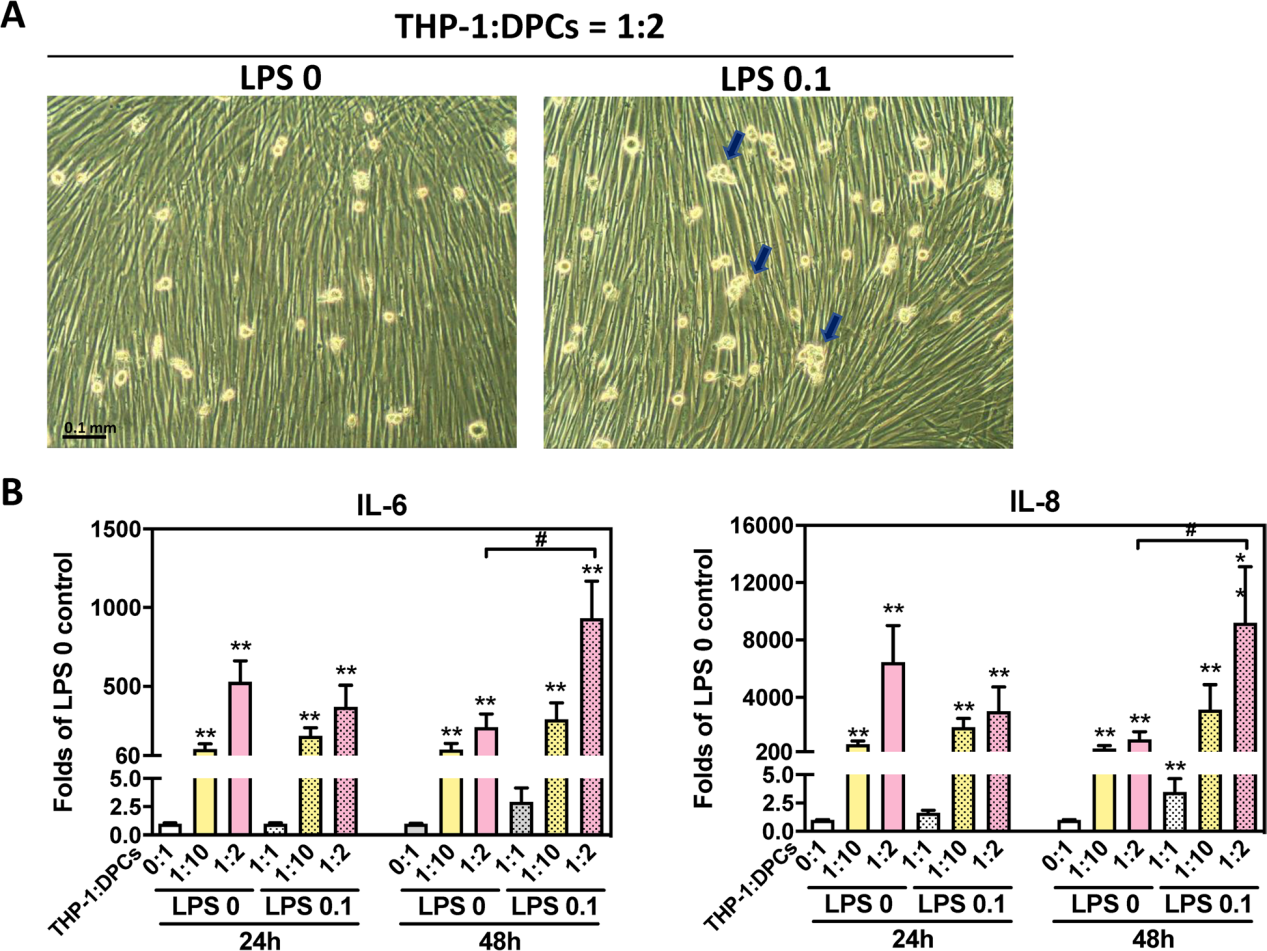
THP-1 enhanced more inflammatory response than LPS in the coculture model. DPCs were pretreated with 0 and 0.1 μg/mL LPS for 24 h, and cocultured with different ratios of THP-1 with 0.1 μg/mL LPS in THP-1 medium. **A** Cell morphology was photographed after 48 h. THP-1 cells were clustered in 0.1 μg/mL LPS-treated group. Triangle arrow – clustered THP-1 cells. Magnification × 100. **B** Total RNA was collected after 24 and 48 h. *IL-6* and *IL-8* mRNA expressions were determined by RT-qPCR. Cocultured with THP-1 increased *IL-6* and *IL-8* expressions at 24 and 48 h. 0.1 μg/mL LPS did not affect THP-1 induced *IL-6* and *IL-8* expressions at 24 h. But 0.1 μg/mL LPS increased THP-1: DPCs = 1:2-increased *IL-6* and *IL-8* expression at 48 h. The data were expressed as the mean ± SD of triplicates from three independent experiments. **, *p* < 0.005 compared with THP-1: DPCs = 0:; #, *p* < 0.05 compare with LPS 0 + THP-1: DPCs = 1:2 of each time point. Mann–Whitney test

DPCs pretreated with 0.1 μg/mL LPS for 24 h, and cocultured with different ratios of THP-1. After 48 h, the cell morphology picture showed that THP-1 cells were clustered in 0.1 μg/mL LPS treated group (Fig. 4A). Both THP-1: DPCs = 1:10 and THP-1: DPCs = 1:2 could increase *IL-6* and *IL-8* expressions at 24 and 48 h (*p* = 0.0022, Fig. 4A and B). 0.1 μg/mL LPS treatment did not affect *IL-6* and *IL-8* expression which was enhanced by THP-1 at 24 h. But 0.1 μg/mL LPS could increase *IL-6* and *IL-8* expressions in THP-1: DPCs = 1:2 at 48 h (*p* = 0.0260, Fig. 4A and B).

**Fig. 5:**
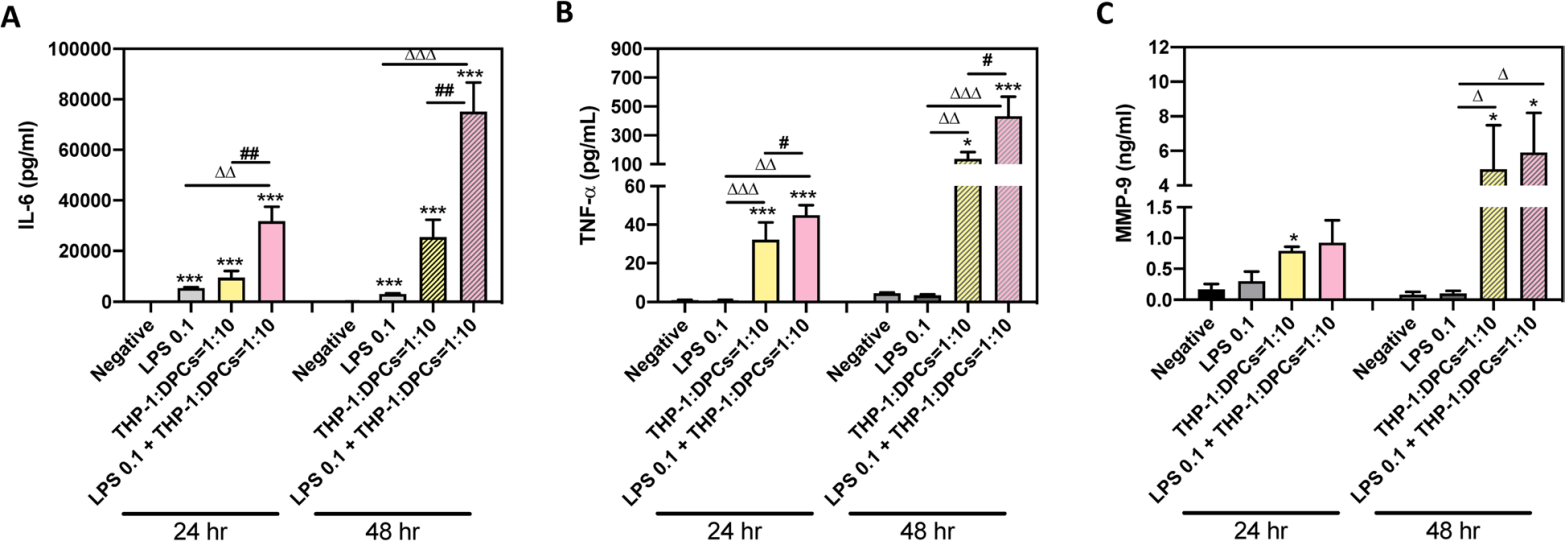
LPS treatment combined THP-1 coculture induced more IL-6, TNF-α and MMP-9 protein expressions. DPCs treated with 0.1 μg/mL LPS alone (labeled as LPS 0.1), coculture with THP-1 cells alone (laneled as THP-1: DPCs = 1:10), or LPS combined THP-1 coculture (labeled as LPS 0.1 + THP-1: DPCs = 1:10). After 24 and 48 h, collect medium and detect IL-6 (**A**), TNF-α (**B**), and MMP-9 (**C**) protein expression by ELISA kits. LPS-combined THP-1 coculture could induce higher IL-6, TNF-α, and MMP-9 protein expression than LPS or coculture with THP-1 treatment. The data were expressed as the mean ± SD of triplicates from two independent experiments. *, *p* < 0.05; ***, *p* < 0.0005 compared with Negative (untreated DPCs); Δ, *p* < 0.05; ΔΔ, *p* < 0.005 compare; ΔΔΔ, *p* < 0.0005 compare with LPS 0.1; #, *p* < 0.05 compare with THP-1: DPCs = 1:10. Mann–Whitney test

DPCs treated with 0.1 μg/mL LPS (LPS 0.1 in Fig. 5) or cocultured with THP-1 (THP-1: DPCs = 1:10 in Fig. 5) for 24 or 48 h could increase IL-6 protein secretion (*p* < 0.0001, Fig. 5A). Moreover, combined LPS treatment and THP-1 coculture could enhance IL-6 expression than LPS treatment or THP-1 coculture (LPS 0.1 + THP-1: DPCs = 1:10 in Fig. 5, *p* = 0.0021 - *p* < 0.0001, Fig. 5A). The protein expression of other immune factors – TNF-α and MMP-9 also demonstrated similar pattern (*p* = 0.0302 - *p* < 0.0001, Fig. 5B and C).

## Discussion

**Fig. 6:**
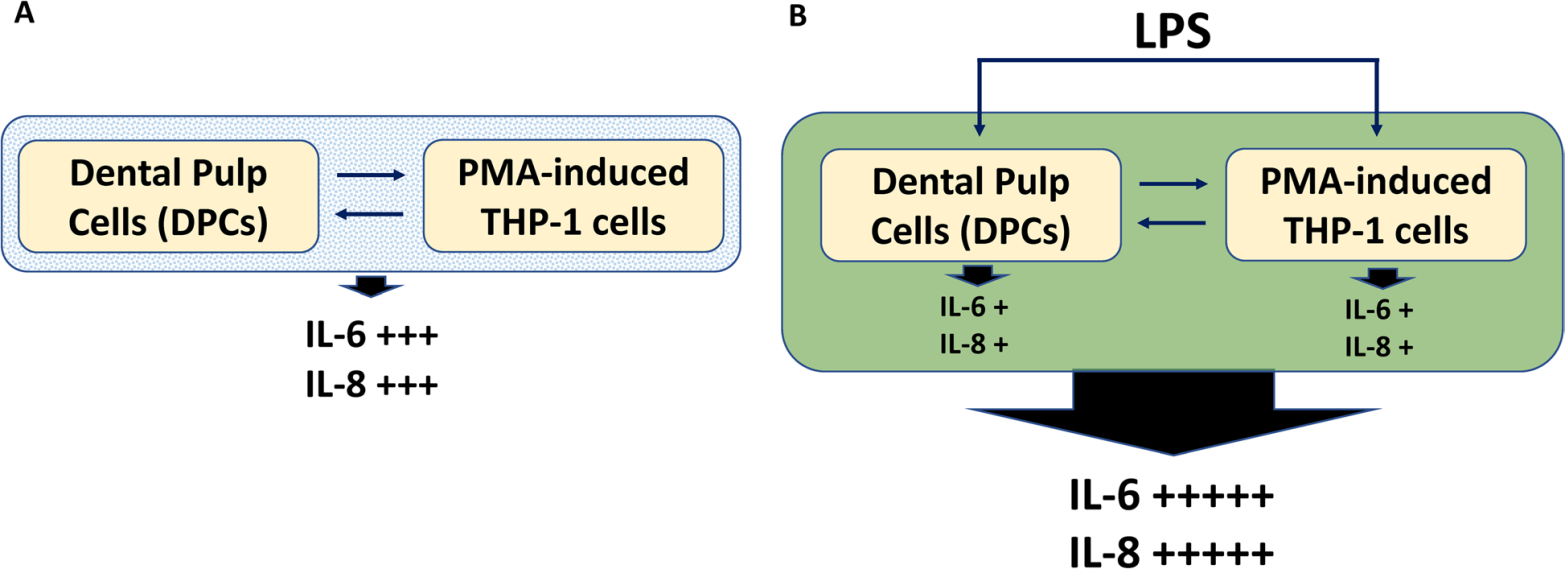
Interactions of DPCs and THP-1 cells under LPS treatment. **A** DPCs cocultured with THP-1 induced *IL-6* and *IL-8* expression without LPS treatment. **B** LPS induced the inflammatory responses of DPCs and THP-1 separately (the increase of *IL-6* and *IL-8*). DPCs cocultured with THP-1 further enhanced *IL-6* and *IL-8* expression with LPS treatment. The interactions between DPCs and THP-1 in inflamed conditions enhance the inflammation to a greater extent

The design of our cocultured model was to depict the mechanisms of the initial steps of pulp inflammation. Following bacteria invasion (the presence of LPS), dental pulp cells produce cytokines (such as IL-6 and IL-8), and inflammatory cells (HL-60, THP-1) migrate to the inflamed pulp area. Then, activated macrophages and inflamed pulp may produce more cytokines and regulate the inflammatory cascades (Fig. 6). The expression levels of IL-6 and IL-8 were used to represent pulpal inflammation, since both cytokines have been shown to be potential biomarkers for irreversible pulpitis [19, 20]. Previously, the coculture models of inflamed dental pulp proposed by Yonehiro et al. were culturing macrophages in the supernatants of inflamed dental pulp cells. Their model demonstrates the cytokines released by dental pulp cells can stimulate macrophages to produce more cytokines (TNF-α) [21]. On the contrary, our model cocultured inflamed dental pulp cells with macrophages in the same inflammatory environment. Therefore, our results have demonstrated that although both pulp cells and inflammatory cells can release cytokines, the inflammation may be modulated by the interactions between pulp, inflammatory cells, and the cytokines. Our coculture models can better mimic histopathological and animal studies and perhaps what happens in the real dental pulp. We could not tell which cell population is causing the changes in the inflammation. Their individual effects and interactions contribute to the whole inflammation outcome.

In traditional cell culture designs, different cell types need to be cultured in different mediums to ensure their viability for further experiments. In the cocultured models of inflamed dental pulp proposed by Yonehiro et al., they use transwell to separate different cells and medium, and their dental pulp cells and macrophages could not have direct contact, which makes them cannot demonstrate the inflammatory cells infiltration to pulp area [21]. We used macrophage medium to culture both dental pulp cells and macrophages in our coculture models. We have confirmed that dental pulp cells cultured in macrophage medium would grow slowly but not yet to the stage of death (Fig. 1C and Fig. S1). In real human dental pulp tissues, the increase of macrophages may be induced by the stimulation of bacteria. Hence, in our cocultured models, different ratios of pulp cells and macrophages were used to represent different severity of pulp inflammation (Fig. 1). The results have shown the higher ratio of the macrophages the more cytokines could be released or expressed (Fig. 2). This may be the novelty of our study. It is worth noting that the expressions of *IL-8* gradually decreased after twelve h in all LPS concentration groups (THP-1 or DPCs alone), but the expressions of *IL-8* still increased after 48 h in a higher ratio of cocultured group (Fig. 3). This may also answer why IL-6, IL-8, and MCP-1 production was mainly enhanced from macrophages instead of LPS in the paper of Yonehiro et al. [21]. Different timing or duration of inflammation may be a key point that needs to be considered in inflammatory study design. The continuing increase of different inflammation markers, such as TNF-*α* and MMP-9, could also be observed in the ELISA level. Furthermore, whether these results implied that the initial pulp inflammation was induced by bacteria but further inflammation was enhanced by the interactions between pulp and inflammatory cells needs further studies. Nevertheless, the inflammatory cytokines IL-6 released from THP-1 were too low and difficult to be observed (Figs. 2, 3, 4 and 5).

Single-cell model studies have demonstrated that inflamed dental pulp cells and macrophages could exhibit inflammatory and repair phenotypes separately [2, 11, 21]. Our results have revealed that the increased folds of *IL-6* and *IL-8* expression in the coculture models were much stronger than the sum of increased folds of *IL-6* and *IL-8* (THP1 or DPCs alone) under LPS treatment. The limitation of our study is that we could not tell whether the increased expressions of *IL-6 and IL-8* were released by the inflamed dental pulp cells or activated macrophages. In a previous paper, inflamed dental pulp cells have been shown to control the differentiation of macrophages, which may produce inflammatory cytokines to affect pulp inflammation and healing [22]. Hence, the increased secretion of cytokines from pulp cells or macrophages may result in different inflammatory pathways. Understanding the pathways may help us to control inflammation more effectively. These may not be revealed according to our study design. Nevertheless, the beauty of coculture models is to depict the interactions of different cells, and perhaps it may be closer to clinical conditions in which inflammation is the outcome of interactions among multiple kinds of cells [6].

Coculture macrophages and dental pulp cells in the presence of LPS might produce even more cytokines, and these effects became more obvious in a longer culturing time (Figs. 4, 5 and 6). This part of our study better illustrated the initial steps of pulp inflammation. In contrast to the transient changes of both cytokines shown in the single-cell model (Fig. 3A and B), the expression of both cytokines in tissues gradually increases with the development of pulpitis [23], concurring with the extending and synergistic enhancement of cytokine expression in our coculture model (Fig. 3C). Furthermore, the expression level of *IL-6* stimulated by LPS of 0.1 μg/mL in the coculture system was comparable to its expression in THP-1 stimulated by LPS of one μg/mL (Fig. 4B vs Fig. 3A). Thus, in addition to the stimulation of pathogen-associated molecular patterns, i.e., LPS in this research, interplay between DPCs and macrophages is also an important determinant for the regulation of cytokine production during pulpal infection. Among these two cytokines, IL-8, a chemoattractant of both neutrophils and T lymphocytes [24], was reported to be strongly expressed by macrophages and lymphocytes but scarcely in fibroblasts in pulp tissues with features of chronic inflammation [25]. IL-6, another tested cytokine, has been known for its broad regulatory effects on immunity, including context-dependent pro−/anti-inflammatory properties of innate responses, regulation of T-cell activities and controls of B-cell survival, expansion and maturation [26]. Therefore, the prolonged production of both cytokines at maximal levels in our coculture model might reflect that the interaction between DPCs and macrophages not only benefits initiating and modulating acute inflammation, but also developing and shaping chronic inflammation in the infected pulp. In the models of Giraud et al., they demonstrated that THP-1 adhered to endothelial cells and then migrated [3], and this may represent the other part of the initial steps [1]. How to connect our study to their design to complete the whole inflammatory story needs to be explored in the future.

Given that the primary objective of vital therapy is to preserve pulp health by impeding inflammation progression, it is imperative to delve deeper into the management of inflamed pulp and promote its healing [27]. Potential strategies, such as anti-cytokine therapies, could pave the way for innovative clinical treatment protocols. For instance, IL-6 is not only produced by pulp cells but also by dendritic cells. These dendritic cells interact with neutrophils and macrophages, orchestrating a complex immunoregulatory response during inflammation [28, 29]. Therefore, a comprehensive understanding of pulp inflammation necessitates the inclusion of a broader spectrum of immune cells in upcoming coculture model studies. Our data of HL-60 showed the possibility of including neutrophils in our future coculturing models.

## Conclusion

Coculturing dental pulp cells and macrophages in the presence of LPS further enhances the expressions of *IL-6* and *IL-8*. These findings may clearly illustrate the initial steps of pulp inflammation which are interplayed by multiple types of cells. This may better help clinical dentists to understand inflammatory mechanisms in real dental pulp. How to apply our coculture models to mimic clinical treatment procedures such as vital pulp therapy (VPT) deserves studying in the future.

### Supplementary Information

**Additional file 1.** 

## Data Availability

The datasets used and/or analysed during the current study are available from the corresponding author on reasonable request.

## Abbreviations

DPCs: dental pulp cells
IL-6: interleukin-6
IL-8: interleukin-8
LPS: lipopolysaccharide
LTA: lipoteichoic acid
PMA: phorbol-12-myristate-13-acetat
RT-qPCR: quantitative reverse transcription
RT-qPCR: tumor necrosis factor-α
MMP-9: matrix metallopeptidase 9
VPT: vital pulp therapy
