# Supplementary material for: Dental pulp cells cocultured with macrophages aggravate the inflammatory conditions stimulated by LPS
Source: BMC Oral Health. 2023 Dec 9;23:991. doi: 10.1186/s12903-023-03625-4 (PMC10710708; doi:10.1186/s12903-023-03625-4)
Supplement: Supplementary file 1 — Additional file 1. [file 12903_2023_3625_MOESM1_ESM.docx]

**Supporting Information**


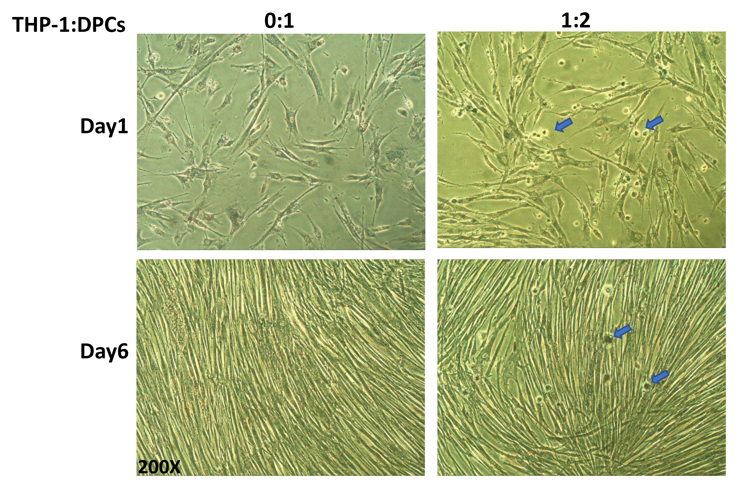


**Figure. S1 The coculture model did not affect the viability of DPCs and THP-1 cells.**

The morphologies of cocultured DPCs and THP-1 cells in the MTT assay. Triangle arrow – insoluble formazan product of THP-1.


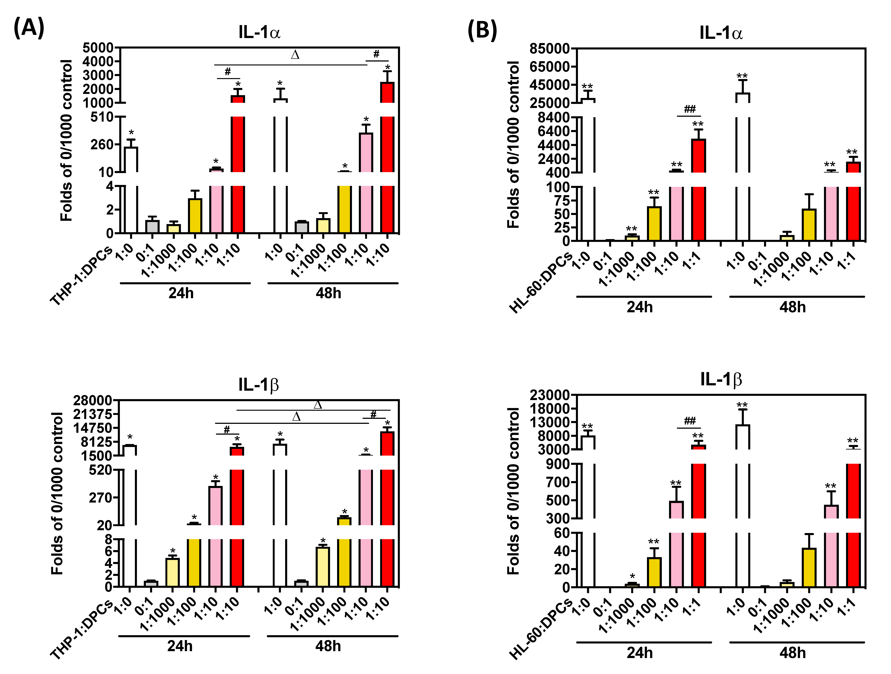


**Fig. S2 Cocultured with 100:1000 ratio of THP-1 cells successfully enhanced *IL-1α* and *IL-1β* expressions.**

DPCs were cocultured with different ratios of THP-1 (A) or HL-60 (B) cells with 100nM PMA and then extracted total RNA after 24 and 48 h. *IL-1α* and *IL-1β* mRNA expression were determined by RT-qPCR. (A) Both THP-1: DPCs = 1:10 and THP-1: DPCs = 1:1 increased *IL-1α* and *IL-1β* expression at 24 and 48 h. The data were expressed as the mean ± SD of triplicates from 3 independent experiments. *, p < 0.05 compared with THP-1: DPCs = 0:1 untreated control of each time point; #, p < 0.05 compared with THP-1: DPCs = 1:10 of each time point; Δ, p < 0.05 compare with 24 h of each ratio group. Mann–Whitney test. (B) Both HL-60: DPCs = 1:10 and HL-60: DPCs = 1:1 increased *IL-1α* and *IL-1β* expression at 24 and 48 h. The data were expressed as the mean ± SD of triplicates from 3 independent experiments. *, *p* < 0.05; **, *p* < 0.005 compared with HL-60: DPCs = 0:1 untreated control of each time point; ##, *p* < 0.005 compared with HL-60: DPCs = 1:10 of each time point. Mann–Whitney test.
